# Static Posturography and Falls According to Pyramidal, Sensory and Cerebellar Functional Systems in People with Multiple Sclerosis

**DOI:** 10.1371/journal.pone.0164467

**Published:** 2016-10-14

**Authors:** Alon Kalron, Uri Givon, Lior Frid, Mark Dolev, Anat Achiron

**Affiliations:** 1 Department of Physical Therapy, School of Health Professions, Sackler Faculty of Medicine, Tel-Aviv University, Tel-Aviv, Israel; 2 Multiple Sclerosis Center, Sheba Medical Center, Tel Hashomer, Ramat-Gan, Israel; 3 Motion Analysis Laboratory, Sheba Medical Center, Tel Hashomer, Ramat-Gan, Israel; 4 Sackler Faculty of Medicine, Tel-Aviv University, Tel-Aviv, Israel; Tokai University, JAPAN

## Abstract

Balance impairment is common in people with multiple sclerosis (PwMS) and frequently impacts quality of life by decreasing mobility and increasing the risk of falling. However, there are only scarce data examining the contribution of specific neurological functional systems on balance measures in MS. Therefore, the primary aim of our study was to examine the differences in posturography parameters and fall incidence according to the pyramidal, cerebellar and sensory systems functional systems in PwMS. The study included 342 PwMS, 211 women and mean disease duration of 8.2 (S.D = 8.3) years. The study sample was divided into six groups according to the pyramidal, cerebellar and sensory functional system scores, derived from the Expanded Disability Status Scale (EDSS) data. Static postural control parameters were obtained from the Zebris FDM-T Treadmill (zebris^®^ Medical GmbH, Germany). Participants were defined as "fallers" and "non-fallers" based on their fall history. Our findings revealed a trend that PwMS affected solely in the pyramidal system, have reduced stability compared to patients with cerebellar and sensory dysfunctions. Moreover, the addition of sensory impairments to pyramidal dysfunction does not exacerbate postural control. The patients in the pure sensory group demonstrated increased stability compared to each of the three combined groups; pyramidal-cerebellar, pyramidal-sensory and pyramidal-cerebellar-sensory groups. As for fall status, the percentage of fallers in the pure pyramidal, cerebellar and sensory groups were 44.3%, 33.3% and 19.5%, respectively. As for the combined functional system groups, the percentage of fallers in the pyramidal-cerebellar, pyramidal-sensory and pyramidal-cerebellar-sensory groups were 59.7%, 40.7% and 65%, respectively. This study confirms that disorders in neurological functional systems generate different effects on postural control and incidence of falls in the MS population. From a clinical standpoint, the present information can benefit all those engaged in physical rehabilitation of PwMS.

## Introduction

Multiple sclerosis (MS) is a chronic, autoimmune, degenerative disease of the central nervous system causing progressive disability in young adults [1]. Subsequently, poor postural control occurs, which is considered one of the most disabling symptoms of the disease. Posture deterioration negatively effects mobility and independence, leading to falls and injuries, adversely affecting the overall quality of life [2]. This deterioration, appearing in people afflicted with multiple sclerosis (PwMS) and with minimal or no clinically assessable impairments [3], becomes more pronounced with significant disease progression [4].

Postural control is a complex skill based on interaction of the visual, somatosensory and vestibular systems, which are frequently impaired in PwMS [5]. Many clinical balance assessment tools are employed in PwMS (e.g the Berg Balance Scale, Functional Reach Test, Tinetti Performance-Oriented Mobility Test, etc) [6]. Nevertheless, it is widely accepted that instrumented tools such as posturography obtain more accurate measurements of postural stability in PwMS [7].

In our previous report, we demonstrated that posturography measures are related to the neurological disability level according to the Expanded Disability Status Scale (EDSS) [8]. While there were non-significant differences in the center of pressure (CoP) trajectories at the lower end of the MS disability scale, a 2 to 3- fold increase in moderate disabled MS patients was noted. Our data add to previous reports [9,10] indicating that posturography measures can be useful in monitoring the progression of MS as well as assessing therapeutic outcomes.

However, some queries remain unanswered as to posturography measures in PwMS. For instance, there are only scarce data examining the contribution of specific neurological functional systems on the CoP movement and sway rate during the stance position [11,12]. Recently, Behrens et al examined static posturography in 90 PwMS and found that the level of impairment in the cerebellar and pyramidal functional systems was correlated with an increased sway rate (Spearman's Rho = 0.499, *P-*value<0.001; Spearman's Rho = 0.211, P-value = 0.047, respectively).

Nevertheless, there were several limitations in Behrens’ study. The impact of damage in more than one neurological system on postural control was not examined, thus preventing the ability to distinguish the impact of the pyramidal, cerebellar and sensory neurological functional systems on static postural control. Furthermore, no information (according to the PubMed database) exists on the associations between posturography measures, neurological functional systems and falls in the MS population. Ultimately, new data on these issues could expand our understanding of the neurological mechanisms involved in balance impairments and accidental falls in PwMS and promote new and improved balance rehabilitation programs for the MS community.

In our recent publication, we reported on spatio-temporal parameters of gait according to neurological subcategories in PwMS. We demonstrated that pyramidal disorders are the main contributors of gait impairments [13]. In the present study, we continue this line of research by examining the differences in posturography parameters and fall incidence according to the pyramidal, cerebellar and sensory systems functional systems in a relatively large sample pool of PwMS.

## Materials and Methods

### Study design and participants

The current study design was cross-sectional. We evaluated retrospective data collected from the Multiple Sclerosis Center, Sheba Medical Center, Tel Hashomer, Israel’s computerized database, documenting demographic and clinical data of all MS patients followed at the Center from January 2012 through May 2016.

A computerized questionnaire was employed to select patients according to the following inclusion criteria: (1) a neurologist-confirmed diagnosis of definite MS according to the revised McDonald criteria [14]; (2) a completed fall status questionnaire and that the patient had undergone a static posturography test between January 2012 and May 2016; (3) the patient was relapse-free for at least 30 days prior to testing; (4) the patient was not participating in any balance rehabilitation program at the time of measurement. Exclusion criteria included: (1) orthopedic disorders that could negatively affect balance; (2) pregnancy; (3) blurred vision; (4) cardiovascular and/or respiratory disorders; and (5) treatment with steroids due to relapse.

The integrity of the data registry was evaluated by a computerized logic-algorithm-questioning process, identifying data entry errors. The study was approved by the Sheba Medical Center Research Ethics Committee (Ethics Ref: 5596-08/244811) confirming extraction of demographical, clinical and posturography data for analysis and full exemption of written or verbal consent from the study participants. Therefore, the individual data will not be made available in order to protect the participants’ identity.

### Expanded Disability Status Scale (EDSS) functional systems

The EDSS, an accepted method of quantifying disability in MS is an eight-function system scale monitoring motor, sensory, cerebellar, brain stem, visual, bowel and bladder, pyramidal and other functions. Each domain is graded from 0 = no disability to 5 or 6 = maximal disability [15]. According to the score achieved from each functional system, an integrated score between 0 = normal examination and 10 = death from MS is derived.

PwMS were divided into six groups, identical to the groups presented in our recent report on spatio-temporal parameters of gait according to neurological functional systems in MS [13]. Classification was determined according to the scores of the pyramidal, cerebellar and sensory functional systems, derived from the EDSS data. When either the pyramidal, cerebellar or sensory domain was ≥2 and the other two domains were either 1 or 0, the patient was included in the relevant group, namely, the pure pyramidal, cerebellar or sensory groups. Patients with a grade of ≥2 in the pyramidal and cerebellar domains were defined as the pyramidal-cerebellar group. A similar approach was established in terms of the sensory and pyramidal domains; patients with a grade of ≥2 only in the pyramidal and sensory domains were defined as the pyramidal-sensory group. Patients with a score of ≥2 in all three domains were defined as the pyramidal-cerebellar-sensory group. Definition of the study groups are summarized in Table 1.

**Table 1:** Definitions of the study groups.

Study groups	EDSS functional system score		
	Pyramidal	Cerebellar	Sensory
Pyramidal	≥2	0 or 1	0 or 1
Cerebellar	0 or 1	≥2	0 or 1
Sensory	0 or 1	0 or 1	≥2
Pyramidal-Sensory	≥2	0 or 1	≥2
Pyramidal-Cerebellar	≥2	≥2	0 or 1
Pyramidal-Cerebellar-Sensory	≥2	≥2	≥2

### Posturography

Static postural control parameters were obtained from the Zebris FDM-T Treadmill data (Zebris^®^ Medical GmbH, Germany) taken at the Center of Advanced Technologies in Rehabilitation, Sheba Medical Center, Israel. A description of the Zebris treadmill is detailed in our previous report on postural control, falls and fear of falling in PwMS [16].

A set of outcome measures taken from the CoP data were:

1. the ellipse sway area (mm^2^), defined as a 95% confidence ellipse for the mean of the CoP anterior, posterior, medial and lateral coordinates.
2. the CoP path length (mm), defined as the absolute length of the CoP path movements throughout the testing period.
3. the sway rate (mm/s), defined as the mean speed of movement of the CoP throughout the testing period. Sway rate = CoP path length/time.

Each participant completed a sequence of three consecutive postural control tests under two different task conditions with a 1-minute break between tasks. Each task was repeated three times for 20-s, followed by a 30-s rest period with:

1. Eyes open: Participants stood barefoot on the treadmill belt (a 10 cm gap between heels, in a 5° toe-out position), in an upright static position with arms resting at their sides. They were instructed to maintain their posture as steady as possible while visually focusing on a dot marked 1m located directly in front of them.
2. Eyes closed: Identical conditions to eyes open but with eyes closed.

The scores for each posturography outcome were calculated as the mean value of the three tests. The rationale as to the current study’s CoP sampling design was discussed in our previous report [16]. Moreover, we calculated the Romberg ratio in the traditional manner according to the following formula: sway with eyes closed/ sway with eyes open [17]. The Romberg ratio is used to assess visual dependency in postural control. A score >1.0 indicates a greater amount of postural sway without visibility.

### Fall status

Participants were defined as "fallers" and "non-fallers" based on their fall history. Input of fall history data was recorded when the patient answered the question: "Have you fallen during the past year?" A fall was defined as an event where the participant unintentionally came to rest on the ground or a lower level [18]. A faller was defined as a participant who had experienced at least two falls during the previous year. Two or more falls were selected since it is questionable whether a single fall clearly classifies an individual as a faller [19].

### Statistics

Descriptive statistics determined the demographic, clinical characteristics and fall status of the study participants. Outliers were determined for each outcome by box plots. Posturography parameters were normally distributed according to the Kolmogorov-Smirnov test. Differences in posturography parameters between PwMS subgroups were determined using the analysis of variance test. A post-hoc Bonferroni test enabled multiple comparisons between the subgroups. For all posturography outcome parameters, the F and P-values are displayed. All analyses were performed using SPSS software (Version 23.0 for Windows, SPSS Inc. Chicago, IL, USA). All reported P-values were two-tailed. The level of significance was set at P ≤0.05.

## Results

The patient group included 342 PwMS, 211 women and 131 men, mean disease duration of 8.2 (S.D = 8.3) years and mean age 46.6 (S.D = 12.0). The mean EDSS for the entire study group was 3.8 (S.D = 1.4, Range 1.5 to 6.5). Pyramidal, cerebellar and sensory impairments were demonstrated in 83.6%, 50.3% and 52.6% patients, respectively. Additionally, 36.8% of the sample was distinguished by an impairment in a single neurological system, while 63.2% were affected by 2 or 3 systems. No differences were observed between the six functional system groups in terms of height (P-value = 0.173) and weight (P-value = 0.130). With respect to disease duration and age, with the exception of the sensory group, no significant differences were found between groups. Individual characteristics and neurological assessment scores are summarized in Table 2.

**Table 2:** Demographic and clinical characteristics of the study group according to the EDSS functional system groups (n = 342).

Variable	Pyramidal (n = 70)	Cerebellar (n = 15)	Sensory (n = 41)	Pyramidal-Cerebellar (n = 77)	Pyramidal—Sensory (n = 59)	Pyramidal—Cerebellar—Sensory (n = 80)	F, *P*-value
Age (yrs)	48.8 (12.2)	43.3 (11.7)	40.6 (12.7)	46.3 (11.9)	46.5 (11.4)	48.7 (11.2)	3.330, 0.116
Gender							
Female	41	11	28	48	41	42	
Male	29	4	13	29	18	38	
Disease duration (yrs)	7.7 (8.4)	5.4 (6.6)	3.8 (6.5)	9.4 (8.4)	8.2 (8.2)	10.3 (8.2)	4.202, 0.001
Height (cm)	169.3 (8.1)	167.1 (8.5)	166.0 (8.7)	168.8 (8.0)	167.4 (9.2)	170.0 (9.1)	53, 0.173
Weight (kg)	73.8 (18.4)	71.9 (25.7)	68.5 (13.6)	66.6 (13.9)	71.5 (15.7)	71.5 (12.3)	1.718, 0.130
EDSS (score)	3.2 (1.3)	2.5 (0.8)	2.5 (0.7)	4.3 (1.1)	3.7 (1.4)	4.7 (1.0)	32.643, >0.001

Scores are presented as mean (SD)

As for fall status, 48.5% (n = 166) of the total sample were categorized as fallers. Fallers had a significant higher EDSS score compared to non-fallers; 4.2 (S.D. = 1.4) vs. 3.3 (S.D. = 1.2), P-Value<0.001. Nonsignificant values were observed between groups in terms of age and disease duration. The percentage of fallers in the pure pyramidal, cerebellar and sensory groups were 44.3%, 33.3% and 19.5%, respectively. As for the combined functional system groups, percentage of fallers in the pyramidal-cerebellar and pyramidal-sensory and groups were 59.7% and 40.7% respectively. The largest proportion of fallers was found in the pyramidal-cerebellar-sensory group, reaching up to 65%. Fall status of the study sample is presented in Table 3.

**Table 3:** Posturography parameters and fall status of the study group according to the EDSS functional system groups (n = 342).

Variable	Pyramidal (n = 70)	Cerebellar (n = 15)	Sensory (n = 41)	Pyramidal-Cerebellar (n = 77)	Pyramidal—Sensory (n = 59)	Pyramidal—Cerebellar—Sensory (n = 80)	F, *P*-Value
Eyes open							
Ellipse area (mm^2^)	118.3 (228.1)	71.1 (71.1)	98.0 (166.2)	217.8 (297.7)	199.7 (278.8)	296.8 (291.6)	5.596, <0.001
CoP path length (mm)	177.9 (113.9)	161.2 (96.3)	139.2 (123.3)	264.4 (167.8)	232.2 (168.2)	356.7 (220.6)	13.908, <0.001
Sway rate (mm/s)	9.0 (5.8)	8.5 (5.0)	7.1 (6.2)	13.5 (8.5)	12.1 (8.4)	18.2 (11.2)	14.026, <0.001
Eyes closed							
Ellipse area (mm^2^)	302.4 (406.4)	131.3 (163.6)	171.9 (303.9)	509.5 (727.8)	491.5 (763.8)	836.6 (825.6)	8.514, <0.001
CoP path length (mm)	349.0 (223.6)	275.4 (232.1)	220.7 (143.8)	507.4 (320.6)	426.3 (308.0)	734.2 (522.1)	9.400, <0.001
Sway rate (mm/s)	17.6 (11.5)	17.3 (15.0)	11.2 (7.3)	26.1 (16.3)	22.7 (15.0)	38.3 (26.2)	17.734, <0.001
Romberg ratio*	1.98 (0.73)	1.63 (0.42)	1.80 (0.84)	2.02 (0.85)	2.05 (1.02)	2.32 (1.31)	2.350, 0.041
Fall status							
Fallers (n, %)	31 (44.3%)	5 (33.3%)	8 (19.5%)	46 (59.7%)	24 (40.7%)	52 (65.0%)	6.141, <0.001
Non-fallers (n, %)	39 (55.7%)	10 (66.6%)	33 (81.5%)	31 (40.3%)	35 (59.3%)	28 (35.0%)	

Scores are presented as mean (SD),

*Romberg ratio = sway with eyes closed/sway with eyes open.

In terms of pure pyramidal, cerebellar and sensory groups, non-significant differences were observed for all posturography parameters (with eyes open and closed) between the three groups. PwMS in the pure pyramidal group scored significantly lower in the sway rate parameter, with eyes open (*P*-Value = 0.02) and closed (*P*-Value = 0.05), compared to patients in the pyramidal-cerebellar group. In contrast, non-significant differences were observed in all posturography parameters between the pure pyramidal group and the combined pyramidal-sensory group. Additionally, non-significant scores were observed between the pure cerebellar group and the combined pyramidal-cerebellar and pyramidal-sensory groups.

Regarding the pure sensory group, the patients demonstrated increased stability according to the CoP path length and sway rate parameters with vision compared to each of the three combined groups: pyramidal-cerebellar (*P*-Value = 0.01, 0.02, respectively), pyramidal-sensory (*P*-Value = 0.04, 0.05, respectively) and pyramidal-cerebellar-sensory (*P*-Value<0.01) groups. Similar results were noted without vision; pyramidal-cerebellar (*P*-Value<0.01), pyramidal-sensory (*P*-Value = 0.05, 0.02, respectively) and pyramidal-cerebellar-sensory (*P*-Value<0.01) groups.

Participants in the pyramidal-cerebellar-sensory combined group demonstrated increased static instability to all posturography parameters compared to the pure pyramidal, pure cerebellar and pure sensory groups (*P*-Value<0.01). Additionally, the combined pyramidal-cerebellar-sensory combined group, scored significantly higher in 5 (out of 6) posturography parameters (the exception was the ellipse area with vision) compared to the combined pyramidal-cerebellar and pyramidal-sensory groups, *P*-Value ranging from <0.01 to 0.02. Non-significant scores were found for all posturography parameters between the pyramidal-cerebellar and pyramidal-sensory combined groups. Posturography scores for the sample pool are provided in Table 3.

**Fig 1:**
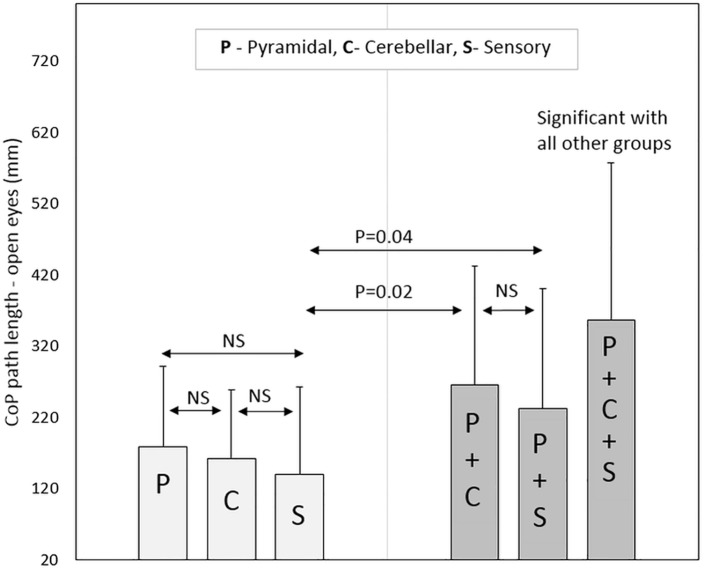
Center of pressure path length (with eyes open) according to neurological functional system groups.

**Fig 2:**
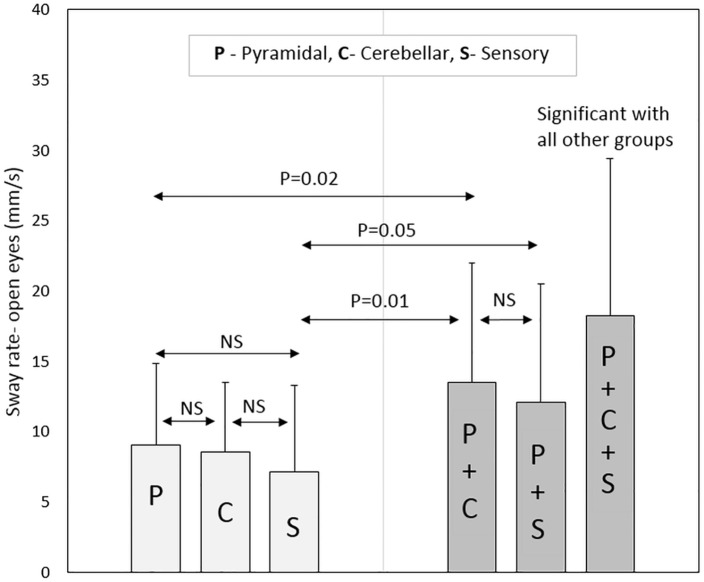
Sway rate (with eyes open) according to neurological functional system groups.

**Fig 3:**
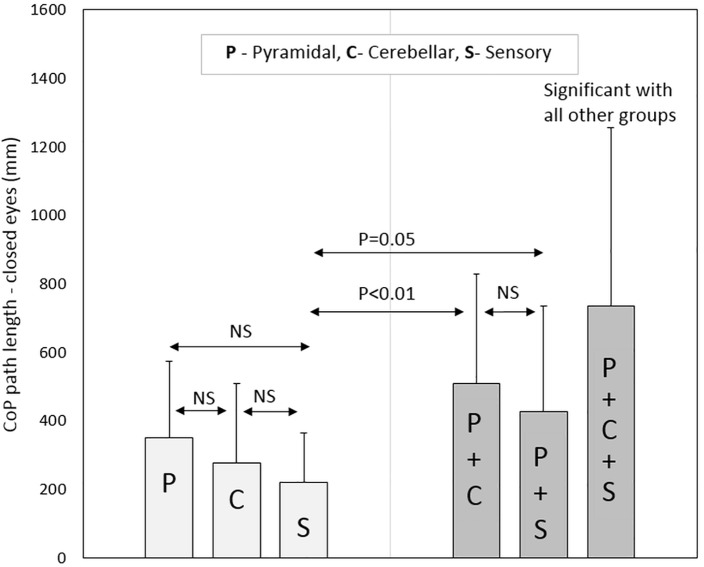
Center of pressure path length (with eyes closed) according to neurological functional system groups.

**Fig 4:**
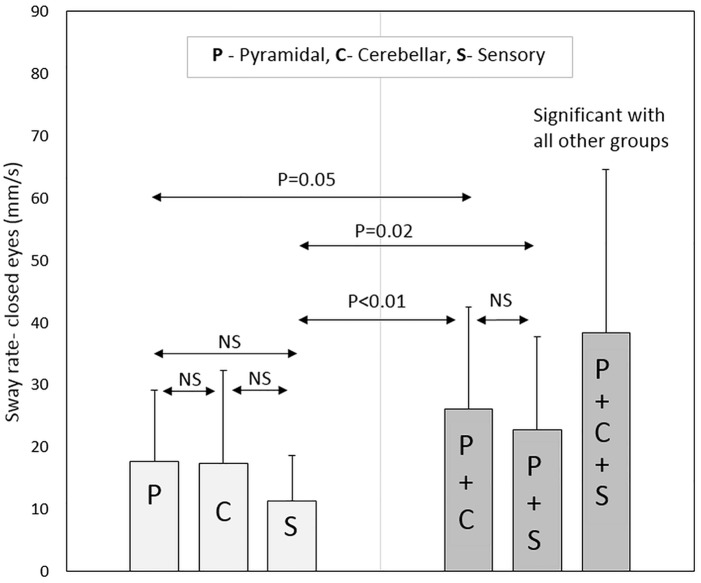
Sway rate (with eyes closed) according to neurological functional system groups.

As for the Romberg ratio, the combined groups had an elevated score compared to the pure groups. However, there was no significant difference between the three pure groups or between the three combined groups. Selected posturography parameters are graphically presented in Figs 1–4.

## Discussion

The primary finding of our study revealed a trend that PwMS affected solely in the pyramidal system have reduced stability and an increased tendency to fall compared to patients with cerebellar and sensory dysfunctions. Moreover, the addition of sensory impairments to pyramidal dysfunction does not exacerbate postural control. In contrast, the addition of cerebellar involvement to pyramidal dysfunction results in a greater sway rate compared to pyramidal dysfunction alone.

Another outcome, that was expected, is that MS participants with damage in three neurological functional systems have decreased stability compared to those negatively affected in one or two functional systems. These main findings concur with our recent report on spatio-temporal parameters of gait in MS according to the EDSS categories [13]. Both studies complement each other as they provide a broad view of the role of neurological functional systems on primary mobility features in MS.

Our results are similar to Behrens et al’s and McLaughlin et al’s previous studies who demonstrated an increase in postural sway in PwMS with pyramidal and cerebellar dysfunction compared to those affected in the visual, brainstem and sensory functional systems [11,12]. However, the major limitation of these reports was that the data analysis did not include combinations of disorders in the three functional systems. We believe that this type of observation is essential in order to attain accurate conclusions. According to the present data, approximately 63% of the sample group were affected in more than one functional system. Based on our literature search, the current study is the first to address this issue in terms of posturography and falls in PwMS.

Interestingly, participants with pure sensory involvement demonstrated increased stability compared to patients affected in two, and obviously, three neurological functional systems. It is obvious that disorders in the somatosensory system results in poor balance. Cameron et al established that slowed somatosensory conduction resulted in imbalance in 10 PwMS [20]. Furthermore, according to the Berg Balance Scale and Timed Up and Go test, sole vibration thresholds were found related to poor balance in PwMS [21]. Conversely, interventions based on stimulation of the sensory system, for example textured insoles, failed to improve gait or balance performance in PWMS [22,23]. We speculate that these inconsistencies are related to the extent of impairment of other functional neurological systems, excluding the sensory system.

Therefore, in cases where PwMS suffer from both pyramidal and sensory dysfunction, focusing solely on the sensory system may not be adequate enough to improve their balance capabilities

The fall data of the study groups reinforces our previous statements on the impact of pyramidal and sensory disorders on posturography measures. According to our results, 44.3% of MS patients in the pure pyramidal group were fallers, compared to 19.5% in the pure sensory group. Furthermore, the percentage of fallers in the combined pyramidal-sensory group was similar to the number in the pure pyramidal group. In contrast, the percentage of fallers in the pyramidal-sensory group was twofold compared to the percentage of fallers in the pure sensory group. In the same context, the percentage of fallers in the pyramidal-cerebellar group was nearly the same in the pyramidal-cerebellar-sensory group. Therefore, we conclude that PwMS who suffer from pyramidal or pyramidal-cerebellar impairments also suffer from poor postural control and are at a high risk of falling. In this situation, the addition of sensory disorders has a limited effect. Clearly, this conclusion is based on posturography measures. Different conclusions may be drawn in cases of clinical balance measures, such as the Berg Balance test and Timed up and Go test, therefore, future studies are encouraged to expand the present knowledge by investigating the scores of various clinical balance tests according to the neurological functional systems.

Our findings can assist professionals involved in the management of balance impairments and falls in PwMS. We feel that intervention programs aimed at improving postural control and reducing falls in PwMS, should be tailored in accordance with neurological systems. Nevertheless, preference should be given to programs addressing significant pyramidal signs such as spasticity, hyperreflexia and muscle weakness, as these symptoms seem to override sensory dysfunction in terms of postural control.

However, to date the literature data on balance problems in individuals with MS has not confirmed this assumption. According to Gunn et al’s systematic review on the effectiveness of interventions to reduce falls and improve balance in PwMS, exercise programs using strength methodologies were found inferior to other intervention programs. However, the sample groups were based on the general disability level, not considering damage in specific neurological functional systems [24].

Limitations of this study include a cross-sectional design. Data was extracted from the Sheba MS computerized database registry, however, possibly some scores were not properly integrated into the database. This occurrence was minimized by a computerized logic algorithm questioning process which identified errors. Secondly, data regarding incidence of falls relied on patients recalling the number of falls which transpired during the past year. There is a likelihood that patients did not accurately report the number of falls due to memory problems. Tertiary, we did not include data of healthy participants, however, this information was provided in our previous report [8]. Finally, we captured static stance which represents a specific feature of balance performance. It would be interesting to examine whether similar findings appear in terms of automatic and anticipatory postural responses.

In summary, this study confirms that disorders in neurological functional systems generate different effects on postural control and incidence of falls in the MS population. While pyramidal involvement is a primary factor negatively affecting static stance in PwMS, patients with sensory impairments have a relatively preserved postural control. Future research studies should investigate whether balance (and fall reduction) interventions, tailored according to the level of involvement of the pyramidal, cerebellar and sensory systems, are more beneficial than traditional rehabilitation.

## Supporting Information

S1 Dataset (SAV)
